# Fall Detection in Individuals With Lower Limb Amputations Using Mobile Phones: Machine Learning Enhances Robustness for Real-World Applications

**DOI:** 10.2196/mhealth.8201

**Published:** 2017-10-11

**Authors:** Nicholas Shawen, Luca Lonini, Chaithanya Krishna Mummidisetty, Ilona Shparii, Mark V Albert, Konrad Kording, Arun Jayaraman

**Affiliations:** ^1^ Max Nader Lab for Rehabilitation Technologies and Outcomes Research Shirley Ryan AbilityLab Chicago, IL United States; ^2^ Center for Bionic Medicine Shirley Ryan AbilityLab Chicago, IL United States; ^3^ Department of Physical Medicine and Rehabilitation Northwestern University Chicago, IL United States; ^4^ Department of Computer Science Loyola University Chicago Chicago, IL United States; ^5^ Department of Bioengineering University of Pennsylvania Philadelphia, PA United States; ^6^ Department of Neuroscience University of Pennsylvania Philadelphia, PA United States; ^7^ Department of Physical Therapy and Human Movement Sciences Northwestern University Chicago, IL United States

**Keywords:** fall detection, lower limb amputation, mobile phones, machine learning

## Abstract

**Background:**

Automatically detecting falls with mobile phones provides an opportunity for rapid response to injuries and better knowledge of what precipitated the fall and its consequences. This is beneficial for populations that are prone to falling, such as people with lower limb amputations. Prior studies have focused on fall detection in able-bodied individuals using data from a laboratory setting. Such approaches may provide a limited ability to detect falls in amputees and in real-world scenarios.

**Objective:**

The aim was to develop a classifier that uses data from able-bodied individuals to detect falls in individuals with a lower limb amputation, while they freely carry the mobile phone in different locations and during free-living.

**Methods:**

We obtained 861 simulated indoor and outdoor falls from 10 young control (non-amputee) individuals and 6 individuals with a lower limb amputation. In addition, we recorded a broad database of activities of daily living, including data from three participants’ free-living routines. Sensor readings (accelerometer and gyroscope) from a mobile phone were recorded as participants freely carried it in three common locations—on the waist, in a pocket, and in the hand. A set of 40 features were computed from the sensors data and four classifiers were trained and combined through stacking to detect falls. We compared the performance of two population-specific models, trained and tested on either able-bodied or amputee participants, with that of a model trained on able-bodied participants and tested on amputees. A simple threshold-based classifier was used to benchmark our machine-learning classifier.

**Results:**

The accuracy of fall detection in amputees for a model trained on control individuals (sensitivity: mean 0.989, 1.96*standard error of the mean [SEM] 0.017; specificity: mean 0.968, SEM 0.025) was not statistically different (*P*=.69) from that of a model trained on the amputee population (sensitivity: mean 0.984, SEM 0.016; specificity: mean 0.965, SEM 0.022). Detection of falls in control individuals yielded similar results (sensitivity: mean 0.979, SEM 0.022; specificity: mean 0.991, SEM 0.012). A mean 2.2 (SD 1.7) false alarms per day were obtained when evaluating the model (vs mean 122.1, SD 166.1 based on thresholds) on data recorded as participants carried the phone during their daily routine for two or more days. Machine-learning classifiers outperformed the threshold-based one (*P*<.001).

**Conclusions:**

A mobile phone-based fall detection model can use data from non-amputee individuals to detect falls in individuals walking with a prosthesis. We successfully detected falls when the mobile phone was carried across multiple locations and without a predetermined orientation. Furthermore, the number of false alarms yielded by the model over a longer period of time was reasonably low. This moves the application of mobile phone-based fall detection systems closer to a real-world use case scenario.

## Introduction

Falls are a common occurrence in the elderly and in people with lower limb amputations. In the elderly, they are the primary cause of injury-related deaths [1], and for people older than 75 years, the estimated percentage who fall is more than 30% per year [2]. Individuals with an amputation, especially elderly with an amputation due to vascular disease, are at a similar or higher risk for falls, with studies reporting that more than 50% of individuals with a unilateral lower limb amputation had fallen in the previous 12 months [3,4]. As such, detecting, understanding, and reacting to falls are of very high importance.

Decreasing response time after a fall and improving fall prevention strategies can dramatically enhance the quality of life for people with lower limb amputations, as well as decrease health care costs. Getting help following an immobilizing fall improves the chance of survival by approximately 50% and increases the likelihood of a return to independent living [5]. Therefore, detecting falls and understanding the environmental circumstances that led to it is crucial for both timely assistance and evaluation of prevention strategies.

Data from real-world fall events are essential for such analyses. However, capturing data from real-world falls is difficult without long-term continuous monitoring [6]. Mobile phones can provide an inexpensive way to detect and measure falls over long time periods [7-10]. All modern mobile phones are equipped with multiple sensors—most notably accelerometers, gyroscopes, barometer, and Global Positioning System (GPS)—which generate a wide range of information regarding the user’s movements and location. Furthermore, mobile phones include memory, computing, and transmission capabilities, which makes them a convenient platform to process the sensors’ data and detect falls [11]. Mobile phones then promise a relatively straightforward generation of large datasets because they can unobtrusively record all the time.

Sensor-based fall-detection algorithms have shown encouraging results in previous studies using young, unimpaired participants performing simulated falls [12,13]. Many of these studies employed threshold-based algorithms, such that a fall is detected if one or more statistical measures (features) computed from the acceleration exceeded a predefined threshold [6,14], whereas some approaches employed machine-learning algorithms, either supervised [15-18] or unsupervised [19]. Such studies showed that simulated falls can be successfully distinguished from daily activities with high accuracy under laboratory-controlled conditions.

Traditionally, studies have used unimpaired individuals for training and testing fall-detection systems. However, a large difference exists between movement patterns of individuals with and without lower limb amputations [20]. Furthermore, most studies have fixed the phone to a specific orientation and location on the body, although phones are carried in multiple locations during everyday use. Common movements, such as taking the phone out of a pocket, could cause large accelerations that may be confused with falls. Therefore, we do not know whether these factors affect the accuracy of a fall-detection algorithm when used with individuals with an amputation.

In this study, we developed a fall-detection classifier that is robust to the previously mentioned sources of error (population, location of the phone, environment) and successfully detect falls in both control (non-amputee) and amputee populations. We collected data from simulated falls and activities from both control volunteers and individuals with transfemoral amputations (TFAs) or above-the-knee amputations in both a laboratory and outdoor environment. To account for the influence of phone location, we collected simulated falls and activities data with the mobile phone carried in three different common locations: in a pouch at the waist, in a pocket, and in the participant’s hand.

## Methods

### Study Design

Data representative of typical activities of daily living and falls was collected from both control participants and participants with a unilateral TFA using a prosthesis. Participants with a TFA were included in the study if they had a unilateral amputation of the lower limb, above or below the knee, within at least 6 months, and if they used either a mechanical or microprocessor-controlled prosthesis on a daily basis. The study was approved by Northwestern University Institutional review board. Written informed consent was obtained from all participants.

Data were collected from the accelerometer and gyroscope sensor of a mobile phone (Samsung Galaxy S4) using the Purple Robot app [21] running on Android 4.4.4. Purple Robot was developed as a research platform for collecting data through hardware sensors on an Android mobile phone. Data from the selected sensors are compiled and transmitted to a remote server for storage and future analysis via Wi-Fi or cellular data connection. The app also allows for some data processing to occur on the phone.

The data sampling rate was approximately 50 Hz and could vary depending on the phone central processing unit usage. Participants carried the phone in three different locations during the data collection: in a pouch worn on the waist, in a pants pocket, or in their hand. Participants were asked to carry the phone in these locations as they would carry their own phone during daily use; as a result, there was some variation in the precise placement of the device (eg, the pocket location included both front and back pockets based on individual preference). During data collection, a researcher annotated the start and end time of each activity or fall with a second mobile phone running Purple Robot.

**Figure 1:**
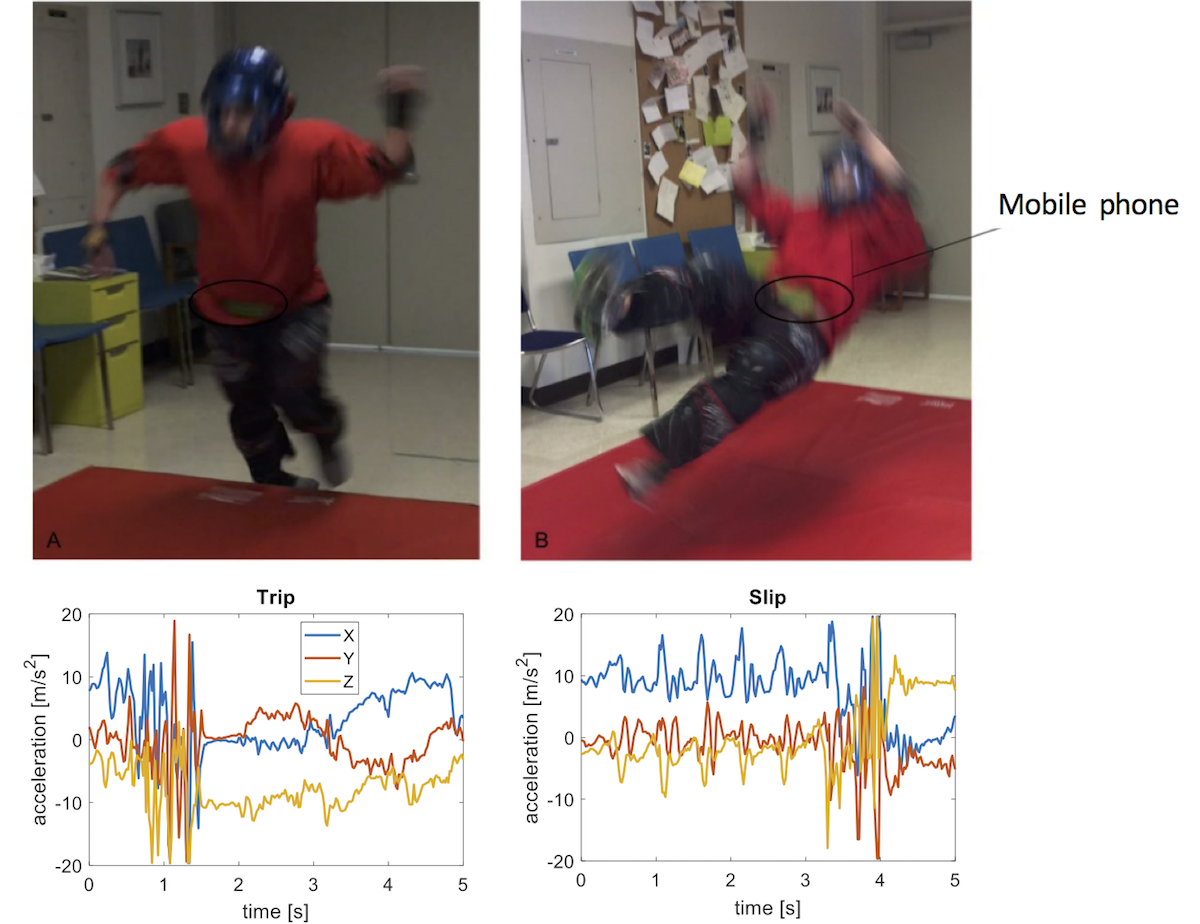
Experimental setup for capturing falls data and detecting falls. A non-amputee volunteer performed a series of falls, including trips (left) and slips (right). A phone was carried in a pouch secured around the waist with a strap belt and in a pants pocket or in hand (not shown). The graphs show example data captured by the phone accelerometer during the two types of falls over a 5 second window.

All participants performed four types of simulated falls: forward (trip), backward (slip), left, and right. Non-amputee participants performed both indoor and outdoor falls, whereas participants with amputation only fell indoors. During all simulated falls, participants fell onto a padded mat (indoor) or grass (outdoor), and wore several layers of padding and guards over the wrists, elbows, knees, and shins to prevent injury. During simulated trips, participants were asked to walk toward the mat and then stumble on the edge as if tripping, then falling forward onto the mat. For slips, participants were instructed to slide one foot out from under them as if slipping and fall backward onto the mat (Figure 1). For lateral falls (left and right), participants were asked to close their eyes and a researcher provided a push to cause the participant to lose their balance and fall onto the mat. The TFA participants were instructed to use whatever protective strategies they might employ in a real fall event, such as turning to avoid landing on their prosthesis. Participants performed each fall type three times for each phone location, for a total of 36 simulated falls.

After completing the falls, participants performed a series of daily activities, again with the phone varied between the three different locations (see Multimedia Appendix 1 for a full list of activities). The different activities performed were sitting, standing, walking, stairs ascent/descent, and lying. During still activities with the phone in hand, participants were asked to use the phone as they usually would (eg, browsing the Internet, checking text messages). In total, approximately 17 hours of labeled data from falls and activities were obtained. In addition, three participants with TFA carried the phone with them for a period between two and seven days, so as to quantify the number of false alarms per day generated by the fall-detection model. No specific instructions were given to the participants, beyond that of carrying the phone in either their pocket or in a pouch around the waist for the majority of their daily routine. All three participants chose to carry the phone in a pants pocket. Over the entire recording period, a mean 213 (SD 182) clips per day (total 2251) exceeded the 2 *g* threshold and were subsequently analyzed.

### Data Preprocessing and Feature Extraction

Data collected by Purple Robot was transmitted to an external server and downloaded for analysis using Matlab 2016b (MathWorks) and Python 2.7. All data were organized into 5-second clips of data for feature extraction. For each fall event, a 10-second long data clip centered on the peak acceleration magnitude (ie, the impact) was extracted; from these clips, ten 5-second long windows were extracted based on a uniform random distribution, such that the beginning of the fall in each 5-second window (clip) can occur with uniform probability in the interval (0 s, 3 s). This was done to provide variety in the location of the falls within a 5-second window.

**Figure 2:**
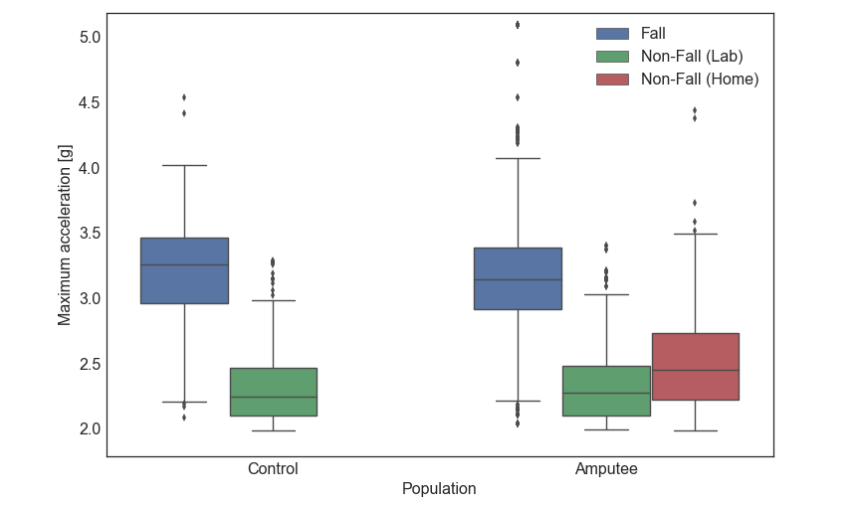
The distribution of maximum acceleration values for falls and non-fall data clips. Values for non-amputee (left) and individuals with TFA (right). Only activity (non-fall) clips with an acceleration greater than 2g were used in the analysis. Peak accelerations from participants’ daily routine (home trial) are also shown for the TFA group. Boxes indicate interquartile (IQR) range; midlines and whiskers represent median and 1.5 IQR, respectively. Individual points denote outliers.

Activities data were generated by taking all data from the beginning to the end of the activities protocol and dividing it into nonoverlapping 5-second windows. Thus, our activity data included postural transitions (eg, sit-to-stand) and phone transitions (eg, pocket-to-hand) that may be confounded with falls by a classifier. We then selected activity clips whose total acceleration (x^2^+y^2^+z^2^) was higher than 2 *g*, which corresponded to the first percentile of the acceleration distribution within the falls (Figure 2). Therefore, only activity clips that included high accelerations and could thus resemble a fall were included in the dataset. In total, 6637 clips (6337 falls, 300 non-falls) of data were obtained from the non-amputee control group and 1815 clips (1537 falls, 278 non-falls) from the TFA group.

After organizing data into the 5-second windows, the accelerometer and gyroscope signals were interpolated to 50 Hz with a cubic polynomial. A total of 40 features were then computed on each axis (x, y, z) and on the resultant vectors (x^2^+y^2^+z^2^) for both the accelerometer and gyroscope signals (Table 1). In training and testing our fall-detection models, all feature vectors derived from activities were labeled as “non-falls” and those derived from simulated falls as “falls.”

### Model Training and Evaluation

**Figure 3:**
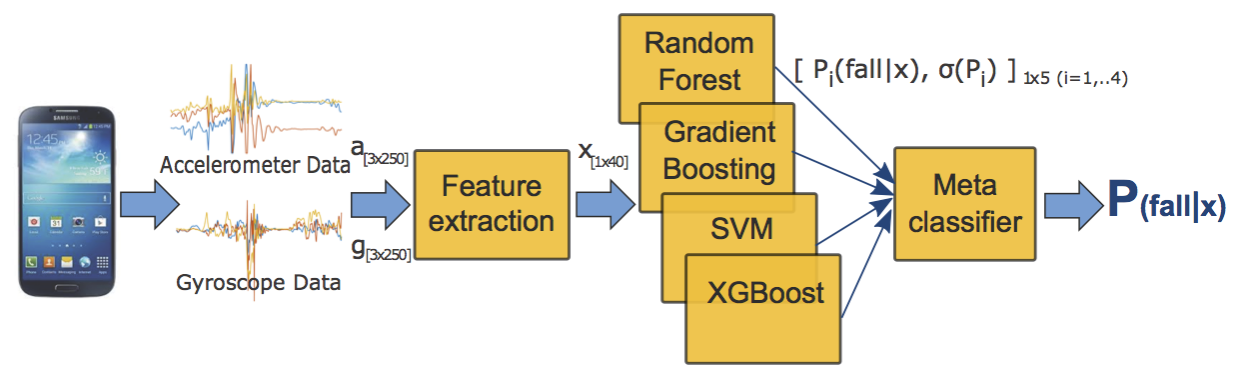
Five-second data clips are recorded from the mobile phone sensors (accelerometer and gyroscope), with each clip yielding a matrix of dimension (3 channels × 250 samples) per sensor. A set of 40 features were calculated from a data clip, and the resulting feature vector x was input to four different classifiers, which were combined through stacking to output the probability of the clip being a fall (see text for details).

We combined the predictions of four different classifiers through stacking [22]: random forest (100 trees), support vector machine [23] (linear kernel, C=1), gradient boosting (100 trees, maximum depth=2), and extreme gradient boosting (XGBoost [24], 150 trees, maximum depth=2, learning rate=0.5, feature subsampling rate=0.6). Each classifier (C1,...,C4) predicts the probability P_i_(fall|x) that the current clip *x* corresponds to a fall, and all the probabilities are combined into a feature vector:

x
_meta_=[P
_1_(fall|x),...,P
_4_(fall|x),σ(P
_i_)]

where *i*=1,...,4, and these probabilities and their standard deviation (σ) are used as input features to a meta-level classifier (logistic regression [25,26]), which learns to combine the individual predictions and outputs the final probability of the clip *x* being a fall (Figure 3).

Classifier performance was evaluated either by leave-one-subject-out cross-validation (LOSOCV) or with an external validation set (see next section). Hyperparameters of the base-level classifiers were tuned using a cross-validated grid search. The primary measures chosen to summarize model performance were sensitivity, specificity, and area under the curve (AUC). Specificity and sensitivity values are reported from the optimal point from the receiver-operator characteristic (ROC) curve, or the point with the greatest sum of sensitivity and specificity. LOSOCV was used for determining the threshold when evaluating the control-to-control model.

As a comparison, we evaluated the performance obtained when using a threshold on a single feature, the maximum acceleration magnitude. This was chosen as a high-performing representative of threshold-based methods because the acceleration magnitude is one the strongest single predictors of a fall and is frequently used [27]. An ROC curve was obtained by measuring the sensitivity and specificity as the threshold on maximum acceleration magnitude was changed, from which we obtained values for AUC, sensitivity, and specificity for this single-feature approach.

**Table 1:** Features computed on each 5-second clip of sensor data (accelerometer and gyroscope) on either the vector resultant or on each axis (x, y, z).

Feature name	Number of features
Mean	1
Median	1
Standard deviation	1
Skewness	1
Kurtosis	1
IQR and derivative of IQR	2
Minimum and derivative of minimum	2
Maximum and derivative of maximum	2
Maximum, minimum, and IQR on each axis (x, y, z)	9
Total per sensor	20

### Effect of Training Population and Phone Location

To determine whether data collected from control (non-amputee) individuals will be effective to detect falls in amputees, we trained and tested models under three training conditions: a model trained and evaluated on control participants using LOSOCV (control to control), a model trained using data from all control participants and evaluated on data from the TFA group (control to amputee), and a model trained and evaluated on TFA participants using LOSOCV (amputee to amputee). Each model was trained and evaluated using data collected in all three phone locations. Furthermore, we assessed the performance of the control-to-amputee model at detecting falls from each individual location (waist, pocket, or hand). Each model was also compared to its corresponding threshold-based version, which represented the baseline performance.

### Measuring Performance at Home

Our model is intended for real-time use on a mobile phone; therefore, we also collected data from TFA participants while they carried the phone in a pocket for at least 2 days. No participant fell during this data collection, so the data represents only non-fall events. This dataset was filtered as before, generating a total of 2467 data clips above the 2 *g* threshold. Resampling and feature extraction were performed as previously to prepare the data for model evaluation. The preceding analysis procedure was repeated, using data collected at home to represent non-falls, rather than daily activities performed in the laboratory.

## Results

A total of 7 amputees (mean 47.4, SD 12.0 years) and 10 control non-amputee participants (mean 24.2, SD 2.2 years) took part to the study. One amputee participant withdrew before performing the outdoor falls protocol and their data was therefore excluded from the analysis. Also, two amputee participants could not complete the entire set of falls because of fatigue. Table 2 describes participant demographics for both groups.

**Table 2:** Demographic information of participants.

Subject ID		Age (years)	Gender	Height (ft/in)	Weight (lbs)	Amputation side	Amputation reason	Type of prosthesis
**Amputee participants**								
	AF004	58	Male	5'10“	203	Left	Trauma	Mechanical
	AF005	51	Female	5'5”	160	Right	Cancer	Microprocessor
	AF006	24	Male	5'10“	205	Right	Cancer	Microprocessor
	AF007	54	Male	6'1”	240	Left	Trauma	Hydraulic
	AF008	37	Female	5'3“	102	Right	Congenital	Mechanical
	AF010	61	Male	5'11”	267	Left	Trauma	Hydraulic
	AF011	47	Male	5'9“	224	Left	Accident	Microprocessor
**Control participants**								
	CF023	23	Female	5'8”	140			
	CF024	24	Female	6'3“	155			
	CF025	24	Female	5'8”	150			
	CF026	23	Female	5'6“	130			
	CF027	23	Female	5'2”	128			
	CF028	25	Male	6'1“	230			
	CF029	27	Female	5'0”	100			
	CF030	29	Female	5'3“	105			
	CF031	21	Male	5'9”	260			
	CF032	23	Male	5'10“	145			

We compared the performance of a fall-detection model trained on data from control individuals with that of a model trained on data from the TFA population. We examined how carrying the mobile phone in different locations affected the accuracy of fall detection. To evaluate false positives during everyday life, we assessed the performance of the model when three individuals with TFA carried the phone for a minimum of 48 hours during their daily routine.

### Effect of Population

**Figure 4:**
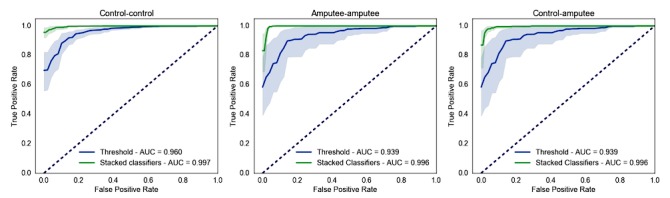
Effect of population on model accuracy. Receiver-operator characteristic curves of fall-detection models based on threshold (blue) or using stacked classifiers (green) trained and tested on data from non-amputee individuals (control-control) and individuals with TFA (amputee-amputee), and trained on non-amputee individuals and tested on TFA data (control-amputee). Shaded areas are 95% confidence intervals from bootstrapping.

Our results indicated that a fall-detection classifier trained using data from control participants was able to reliably separate falls from daily activities in individuals with TFA (Figure 4). The performance of this model (control to amputee: AUC mean 0.996, 1.96*standard error of the mean [SEM] 0.004) was not significantly lower than that of a model trained on TFA individuals (amputee to amputee: AUC mean 0.995, SEM 0.004) (Wilcoxon rank-sum test, z=.40, *P*=.69). Fall-detection accuracy for a model trained and tested on non-amputee individuals yielded similar performance (control to control: AUC mean 0.997, SEM 0.003) (z=.65, *P*=.52). Stacking classifiers outperformed the threshold-based classification models in both populations (control to control: z=3.63, *P*<.001; amputee to amputee: z=1.92, *P=*.06; control to amputee: z=2.08, *P=*.04). Table 3 summarizes the fall detection results obtained with each model. Therefore, detecting falls in individuals with TFA could be achieved by only using training falls and activity data from non-amputee individuals.

**Table 3:** Summary results for models trained and tested on each population (control or amputee). Sensitivity and specificity values represent the optimal point of the ROC curve.

Method and performance metric		Model, mean (1.96 SEM)		
		Control-control	Control-amputee	Amputee-amputee
**Stacking**				
	AUC	0.997 (0.003)	0.996 (0.004)	0.995 (0.004)
	Sensitivity	0.979 (0.022)	0.989 (0.017)	0.984 (0.016)
	Specificity	0.991 (0.012)	0.968 (0.025)	0.965 (0.022)
**Threshold-based**				
	AUC	0.960 (0.020)	0.939 (0.059)	0.939 (0.059)
	Sensitivity	0.915 (0.040)	0.878 (0.097)	0.878 (0.097)
	Specificity	0.927 (0.045)	0.922 (0.045)	0.922 (0.045)

### Effect of Location

**Figure 5:**
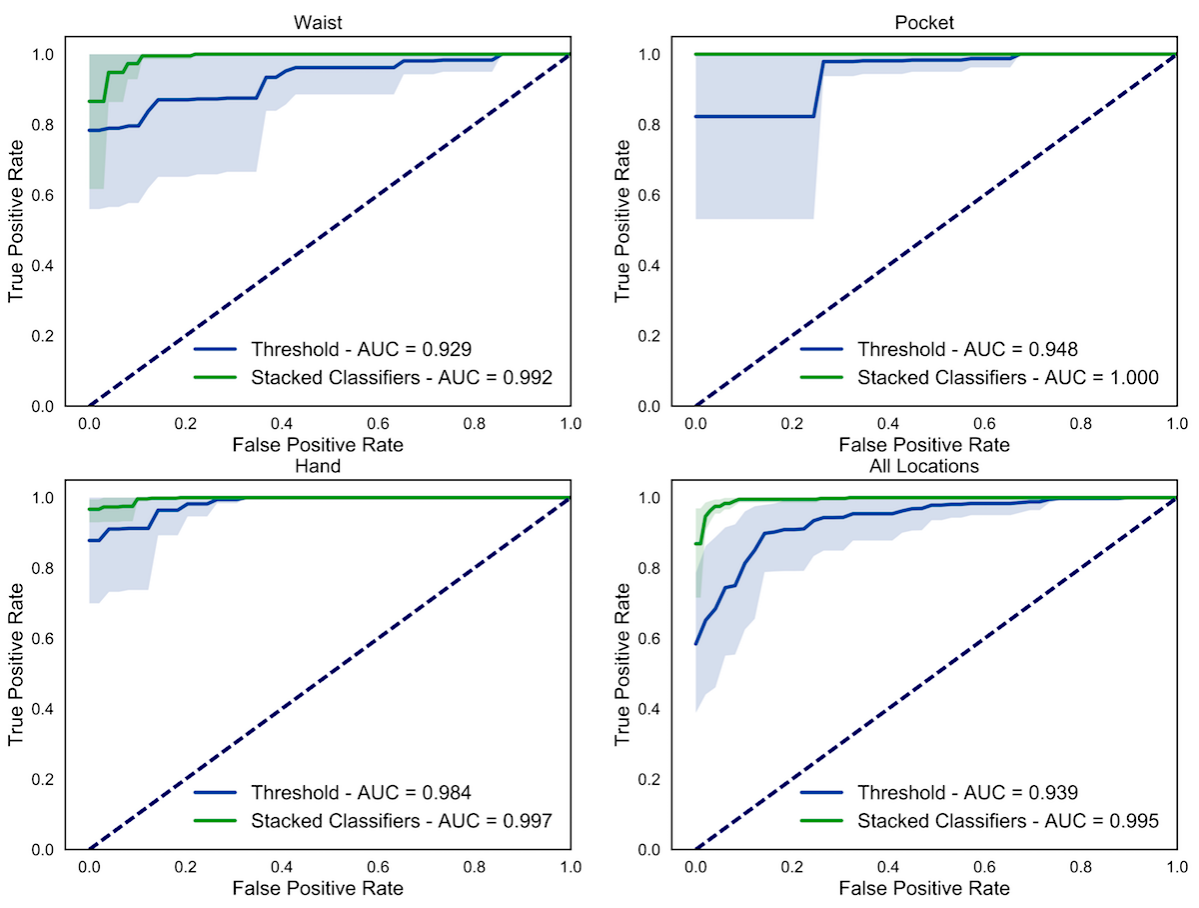
Effect of phone location on fall-classification accuracy. Receiver-operator characteristic curves for the control-amputee model organized by test location (green: stacked classifiers; blue: threshold model). Shaded areas are 95% confidence intervals.

To assess how the location where the phone was carried affected fall-detection accuracy, we examined the performance of the control-amputee model for each location separately (Figure 5). Carrying the phone in the pocket yielded the highest AUC (mean 1.000, SEM 0.000) followed by hand (mean 0.997, SEM 0.003) and waist (mean 0.992, SEM 0.012); however, no statistically significant differences were found between these values (z=0.37-1.47, *P*=.14-.72). Our stacked classifier model showed less intersubject variability than threshold approaches. A summary of results is reported in Table 4. Regardless of the location, the AUC values suggest good algorithm performance for the stacked classifiers.

**Table 4:** Summary results for the control-to-amputee model tested on in-laboratory data organized by phone location.

Method and performance metric		Mobile phone location, mean (1.96 SEM)			
		Waist	Pocket	Hand	All
**Stacked classifiers**					
	AUC	0.992 (0.012)	1.000 (0.000)	0.997 (0.003)	0.996 (0.004)
	Sensitivity	0.990 (0.017)	1.000 (0.000)	0.989 (0.013)	0.989 (0.017)
	Specificity	0.982 (0.033)	1.000 (0.000)	0.980 (0.036)	0.968 (0.025)
**Threshold**					
	AUC	0.929 (0.097)	0.948 (0.065)	0.984 (0.027)	0.939 (0.059)
	Sensitivity	0.932 (0.087)	0.979 (0.036)	0.995 (0.010)	0.878 (0.097)
	Specificity	0.915 (0.112)	0.934(0.115)	0.939 (0.092)	0.922 (0.045)

### Home Data Analysis

A fall-detection model can be effectively deployed outside of a laboratory setting if the number of false alarms generated is small. Therefore, we tested our model on data from three individuals with TFA who, following the experimental session, carried the mobile phone with them during their daily routine for a period between 2 and 7 days. All participants chose to carry the phone in their pocket and were not given any indication on how to carry the phone otherwise. The participants did not experience a fall during the recording period. Testing the model during the home period allowed us to meaningfully assess the false positive rate.

**Figure 6:**
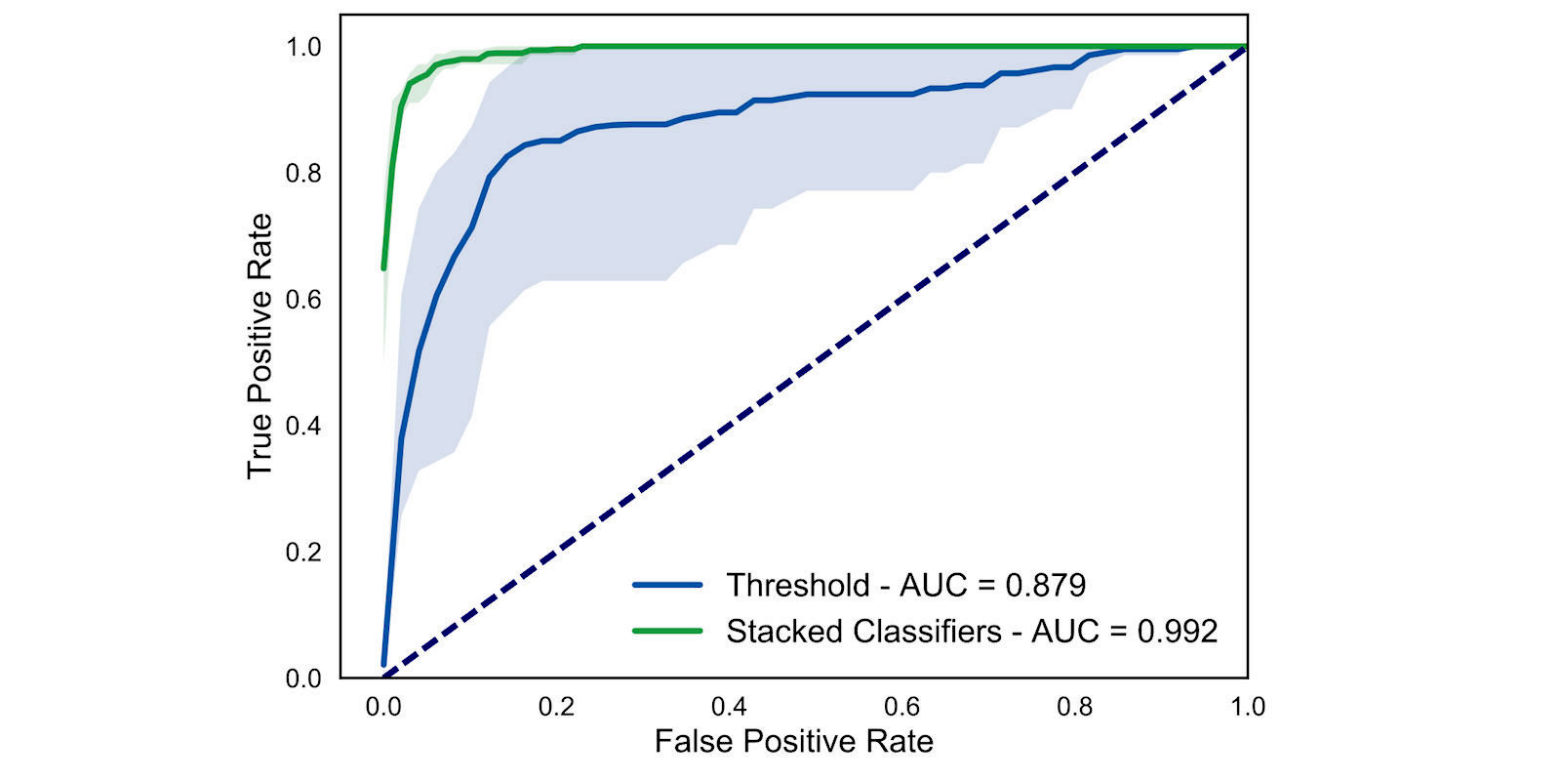
Fall-detection performance on home data. Receiver-operator characteristic curve averaged across the three amputee participants. Data include both the participants’ daily routine data and the in-laboratory falls. Shaded areas are 95% confidence intervals.

Performance of our model on the home data was comparable to performance obtained on laboratory data (control-to-amputee model, Figure 6 and Table 5; z=1.03, *P*=.30), with a mean AUC across the three participants of 0.992 (SEM 0.001); sensitivity: mean 0.970, SEM 0.021; specificity: mean 0.950, SEM 0.016. The stacked classifiers model also performed better than the threshold method (z=1.96, *P*=.05), with all results reported in Table 5. We also calculated the false alarms rates for these three participants: at a sensitivity of 90%, there were 1.0, 4.6, and 1.1 false alarms per day (mean 2.2, SD 1.7), whereas for the threshold approach, the false alarm rates were 4.9, 357.0, and 4.4 per day (mean 122.1, SD 166.1), respectively. The high false positive rate for the second participant appears to be the result of both low acceleration magnitude during simulated falls and high acceleration magnitude during at-home activities. Thus, our model can effectively detect falls while keeping its false alarm rate to a reasonable low value when deployed outside of a laboratory-controlled scenario.

**Table 5:** Summary results for each model on home data.

Performance metric	Method, mean (1.96 SEM)	
	Stacked classifiers	Threshold
AUC	0.992 (0.001)	0.879 (0.121)
Sensitivity	0.970 (0.021)	0.842 (0.188)
Specificity	0.950 (0.016)	0.844 (0.011)

## Discussion

### Principal Results

We developed a fall-detection classifier using data collected from the inertial sensors of a mobile phone, carried by non-amputee individuals and by individuals with a TFA. In order to mimic a naturalistic setting, phones were carried in three common locations (pouch, pocket, or hand) and without standardizing the orientation of the phone. We observed no significant effect of the population used for training the model on the fall detection accuracy; a model trained on non-amputee data was as accurate as one trained on our pool of individuals with TFA. Therefore, fall detection can be reliably performed in amputee participants using data from non-amputee participants.

Previous studies have generally used validation on non-amputee participants despite the fact that the clinical population is the real target. However, participants with mobility impairments display different movement patterns from unimpaired individuals, which can affect the accuracy of activity recognition classifiers [28-30]. We pursued the possibility that amputee movements during activities and simulated fall events may have been unique enough to suggest population-specific model training. Our results did not show such a dependency, thus suggesting that a fall-detection model trained on non-amputee individuals can generalize to other clinical populations prone to falls.

We collected falls and activity data as the phone was carried across common locations to mimic a naturalistic scenario. Nevertheless, we observed that the peak acceleration from real-world data exceeded the range of accelerations of the in-laboratory activities. This is not surprising because prior studies also found that in-laboratory activities look different from real-world unstructured behaviors [30]. Thus, falls and activity data during natural use of the phone must be collected to build a fall-detection system that can be deployed in an everyday scenario.

We compared a machine-learning model based on 40 features to a threshold-based approach, which used a single feature (maximum acceleration) to detect whether the motion of the phone constituted a fall event. Our model yielded an average of two false alarms per day versus approximately 122 produced by the threshold model. This result is at least as good as current state-of-the-art fall-detection systems based on a waist-mounted wearable accelerometer [6]. Therefore, combining multiple features through machine learning confers a significant advantage to build a robust phone-based fall detection system.

The purpose of this work was to develop a system that can capture real-world falls with high probability, while reducing the number of false alarms. In addition to the inertial measurement unit (accelerometer, gyroscope) data used for the study, mobile phones can also collect location and weather data, as well as responses to survey questions sent to the participants. This data could be uploaded to a remote server for further analysis, including classification of fall types and the activity preceding the fall. This host of information can be used to build better fall monitoring systems, as well as understanding the context where real falls can occur in amputees.

### Limitations

One limitation of this study is the fact that we did not incorporate real-world falls, but rather falls in a controlled setting either initiated by the participant (for slips and trips) or induced by pushing (for lateral falls). Our goal was to collect falls data that approximate real falls, while being practical and safe for our participants. Real falls may have a different movement pattern than simulated falls [31], and algorithms developed on simulated falls can fail when tested on real falls [6]. We will acquire real-world falls data for future work by letting participants carry the mobile phones and report the natural falls when they occur using the Purple Robot app; this data could then be used to refine the detection algorithm and improve the system reliability over time.

Although two false alarms per day is a reasonably low value, this result might still produce a large number of false positives relative to the total number of falls that can be expected to capture. Additional analysis is necessary to differentiate false positives from actual falls. This could be done by analyzing other sources of information surrounding the fall, such as GPS and activity data. For example, this would distinguish someone who fell and remained on the ground from someone who lost their balance but continued to walk afterwards, or a phone dropped on the ground and then picked up. Such an analysis could be performed either on the phone or on a remote server, depending on its complexity.

A larger feature set or larger clip lengths could also be used to further reduce the number of false positives, and remains to be explored. For example, previous models have used features describing posture and frequency domain features [6,15]. However, the computational complexity has to be balanced against the need for real-time continuous monitoring. Alternatively, a more complex analysis could be also run on a remote server, with the phone filtering for probable falls.

### Conclusions

We developed a machine-learning classifier to detect falls in people with lower limb amputations using data from mobile phone inertial sensors. Our results demonstrate that a classifier trained on falls from non-amputee participants can reliably generalize to other populations while the mobile phone is naturally carried in multiple locations. Our approach yields a significant advantage over a threshold-based classifier because it drastically reduces the number of false alarms, which is arguably necessary for fall-detection system to be of practical use. By applying the techniques used here, along with improvements in battery management, we believe that fall detection comes one step closer to improving the interventions performed after individuals with disabilities experience a fall. Currently, we are in the process of collecting real-world falls in individuals with lower limb amputations to test our fall-detection system in everyday life.

## Appendix

Multimedia Appendix 1 Activity protocol. The set of activities performed by the subjects to mimic daily functional activities.

## Abbreviations

AUC: area under the curve
GPS: Global Positioning System
LOSOCV: leave-one-subject-out cross-validation
ROC: receiver-operator characteristic
SEM: standard error of the mean
TFA: transfemoral amputation
