# Addendum of: Fall Detection in Individuals With Lower Limb Amputations Using Mobile Phones: Machine Learning Enhances Robustness for Real-World Applications

**DOI:** 10.2196/mhealth.9177

**Published:** 2017-12-20

**Authors:** Nicholas Shawen, Luca Lonini, Chaithanya Krishna Mummidisetty, Ilona Shparii, Mark V Albert, Konrad Kording, Arun Jayaraman

**Affiliations:** ^1^ Max Nader Lab for Rehabilitation Technologies and Outcomes Research Shirley Ryan AbilityLab Chicago, IL United States; ^2^ Department of Physical Medicine and Rehabilitation Northwestern University Chicago, IL United States; ^3^ Department of Computer Science Loyola University Chicago Chicago, IL United States; ^4^ Department of Bioengineering University of Pennsylvania Philadelphia, IL United States; ^5^ Department of Neuroscience University of Pennsylvania Philadelphia, IL United States; ^6^ Department of Physical Therapy and Human Movement Sciences Northwestern University Chicago, IL United States

**Keywords:** fall detection, lower limb amputation, mobile phones, machine learning

The authors of the paper “Fall Detection in Individuals With Lower Limb Amputations Using Mobile Phones: Machine Learning Enhances Robustness for Real-World Applications” (JMIR Mhealth Uhealth 2017;5(10):e151) inadvertently omitted a funding source in the Acknowledgments. Therefore, we would like to change the acknowledgments section of their paper to the following:

The authors are thankful to Mark Begale and Christopher J Karr from CBITs for their technical assistance with the Purple Robot phone app. The authors would also like to thank Dr Saninder Kaur, Kelsey Greenoe, Ashley Adamczyk, and Ryan Griesenauer for their assistance during participant recruitment and data collection. This study was funded by the National Institute of Health – NIBIB grant 5 R01 EB019406-04 and by the Max Näder Rehabilitation Technologies and Outcomes Research Center of the Shirley Ryan Ability Lab (formerly Rehabilitation Institute of Chicago).

The corrected article will appear in the online version of the paper on the JMIR website on December 20, 2017, together with the publication of this correction notice. Because this was made after submission to PubMed Central, the corrected article will also be re-submitted to PubMed Central.

