# Supplementary material for: Fall Detection in Individuals With Lower Limb Amputations Using Mobile Phones: Machine Learning Enhances Robustness for Real-World Applications
Source: JMIR Mhealth Uhealth. 2017 Oct 11;5(10):e151. doi: 10.2196/mhealth.8201 (PMC5656773; doi:10.2196/mhealth.8201)
Supplement: Multimedia Appendix 1 [file mhealth_v5i10e151_app1.pdf]

Supplementary table: Activity protocol. The set of activities performed by the subjects to mimic daily functional activities.

Activity	Phone Location	Duration
Sitting	Hand	60s
Standing	Hand	30s
Walking	Hand	60s
Walking	Pocket	60s
Standing	Pocket	30s
Sitting	Pocket	60s
Sitting	Hand	60s
Standing	Hand	30s
Walking	Hand	60s
Walking	Waist	60s
Standing	Waist	30s
Sitting	Waist	60s
Stairs Descent	Waist/Pocket*	30s
Stairs Ascent	Waist/Pocket*	30s
Lying	Waist	60s
Lying	Hand	60s
Lying	Pocket	60s

* Location for stairs descent/ascent was assigned randomly as waist or pocket
